# Investigation of Clinical, Laboratory, Imaging Findings and Histopathological Features of Patients with Gastric Neuroendocrine Cell Hyperplasia

**DOI:** 10.5152/tjg.2024.22681

**Published:** 2024-02-01

**Authors:** Burcu Çoban, Göksel Bengi, Gözde Derviş Hakim, Şadiye Mehtat Ünlü, Dudu Solakoğlu Kahraman, Funda Barlık, Gamze Çapa Kaya, Müjde Soytürk

**Affiliations:** 1 Division of Internal Medicine, Dokuz Eylül University, İzmir, Turkey; 2 Division of Internal Medicine, Department of Gastroenterology, Dokuz Eylül University, İzmir, Turkey; 3 Division of Internal Medicine, Department of Gastroenterology, Health Sciences University, İzmir Faculty of Medicine, Tepecik Training and Research Hospital, İzmir, Turkey; 4 Department of Pathology, Dokuz Eylül University, İzmir, Turkey; 5 Department of Pathology, Health Sciences University, İzmir Faculty of Medicine, Tepecik Training and Research Hospital, İzmir, Turkey; 6 Department of Radiology, Dokuz Eylül University, İzmir, Turkey; 7 Department of Nuclear Medicine, Dokuz Eylül University, İzmir, Turkey

**Keywords:** Gastric biopsy, neuroendocrine cell hyperplasia, neuroendocrine tumor

## Abstract

**Background/Aims:**

Neuroendocrine cell hyperplasia is a non-neoplastic proliferation of enterochromaffin-like cells and is considered a premalignant lesion because of their potential to progress to neuroendocrine tumor. In this study, we aimed to evaluate the demographic and clinical features, laboratory, radiological and endoscopic findings, gastric biopsy histopathological features, follow-up frequency, and histopathological findings of patients diagnosed with gastric neuroendocrine cell hyperplasia as well as to investigate the factors that play a role in the development of neuroendocrine tumors on the basis of neuroendocrine cell hyperplasia.

**Materials and Methods:**

The study has been conducted in 2 centers with 282 patients that were grouped as those with and without neuroendocrine tumor. Individuals with control endoscopy were separated as those with regression of neuroendocrine cell hyperplasia and those without regression, and the determined parameters were evaluated between the groups.

**Results:**

The most common histological subtype of neuroendocrine cell hyperplasia was linear + micronodular (50.4%). Neuroendocrine tumor developed in 4.3% (12/282) of the patients with neuroendocrine cell hyperplasia after a mean of 36 months. The presence of polyps as confirmed via endoscopy and dysplasia as confirmed via histopathological examination was significantly higher in favor of the group with neuroendocrine tumor (*P* = .01). In patients with neuroendocrine cell hyperplasia regressed and patients in whom it did not regress were examined, the rate of asymptomatic patients and increased sedimentation rate were found in favor of the group that did not regress (*P* = .02 and *P* = .02), but no difference was found in other parameters.

**Conclusion:**

Neuroendocrine tumor development rate was found to be 4.3% in the background of neuroendocrine cell hyperplasia. Two factors predicting progression from neuroendocrine cell hyperplasia to neuroendocrine tumor can be elaborated as the presence of polypoid appearance due to neuroendocrine cell hyperplasia as confirmed via endoscopy and dysplasia as confirmed via histopathological examination.

## Introduction

Neuroendocrine cell hyperplasia (NECH) is a non-neoplastic proliferation of enterochromaffin-like (ECL) cells and is considered a premalignant lesion because of their potential to progress to neuroendocrine tumor (NET). Neuroendocrine cell hyperplasia can be seen in approximately 10% of patients who underwent endoscopy for various reasons.^1,2^

**Main Points**

Neuroendocrine Cell Hyperplasia (NECH) is a premalignant lesion. It is aimed to evaluate the demographic and clinical features, laboratory, radiological and endoscopic findings, gastric biopsy histopathological features, follow-up frequency and histopathological findings of patients diagnosed with gastric neuroendocrine cell hyperplasia.
The most common histological subtype of NECH was linear + micronodular (49.1%).
NET development rate was found to be 3.9% in the background of NECH.
Two factors predicting regression of NECH progression from NECH to NET can be elaborated as presence of polypoid apperance due to NECHpolyps in endoscopy and dysplasia in histopathological examination.

It has been shown that there is a progression from simple hyperplasia to linear and micronodular hyperplasia, then to dysplasia, and ultimately to the development of NET, mainly by the trophic effect of gastrin on ECL cells in the stomach.^2,3^ The coexistence of variable degrees of ECL cell hyperplasia with gastric NETs (gNETs) in hypergastrinemic patients supports the progression of ECL cell hyperplasia to the development of gastric carcinoid through a well-known cascade of events.^4^

Considering the etiology of NET, especially in risky geographical regions, accompanying environmental and personal factors such as age, gender, non-steroidal anti-inflammatory drug (NSAID) use, proton pump inhibitor (PPI) use, smoking and alcohol consumption, accompanying diseases *Helicobacter pylori* infection, Zollinger–Ellison syndrome (ZES), and multiple endocrine neoplasia type 1, and some endoscopic and histopathological findings such as gastric polyp, chronic atrophic gastritis (CAG), intestinal metaplasia (IM), NECH, or neuroendocrine cell dysplasia are thought to play a role in the development of the disease.^5^

Neuroendocrine cell hyperplasia has no distinctive findings in terms of clinical and laboratory features, imaging findings, or endoscopic appearance. However, accompanying NET may present with different clinical pictures depending on the localization of the tumor and the hormone secreted by the functional ones.^6^ The gold standard in the diagnosis of NECH is the histopathological examination of multiple biopsies taken from all lesions and the surrounding mucosa during endoscopy in suspected patients.^7^

Neuroendocrine cell hyperplasia is a premalignant lesion, and intermittent endoscopic biopsy follow-up is recommended.^8^ However, as far as is known, not all hyperplasias progress to tumor; some of them can be reversed by eliminating the predisposing factors, and a few of them can transform to NET after a few years or longer.^9^ There are limited data in the literature that reveal the characteristics of NECH cases and the factors that play a role in the progression from NECH to NET.

The aim of this study was to evaluate the demographic and clinical features, laboratory, radiological and endoscopic findings, gastric biopsy histopathological features, frequency of follow-up, and histopathological findings in the follow-up of NECH patients diagnosed in our centers as well as to investigate the factors that play a role in the development of NET in the background of NECH.

## Materıals and Methods

This study had a retrospective cross-sectional nature. A total of 282 patients who underwent upper gastrointestinal system (GIS) endoscopy between May 1, 2011, and May 1, 2020, in Dokuz Eylül University Medical Faculty Hospital and Tepecik Training and Research Hospital Gastroenterology Department have been enrolled in this study. The ethics committee approval has been granted by Dokuz Eylül University Medical Faculty Ethics Committee on May 11, 2020, with protocol number 5363-GOA. Informed consent has been obtained from all participants.

Age, gender, comorbid diseases (including Hashimoto’s thyroiditis and type 1 diabetes mellitus (T1DM) and other accompanying autoimmune diseases), drugs used (PPI, H2 receptor blockers, aspirin, and NSAID), smoking and alcohol use, family histories, and complaints on admission were recorded.

The most recent routine hemogram and biochemical parameters are as follows: lactate dehydrogenase (LDH), erythrocyte sedimentation rate, C-reactive protein, carcinoembryonic antigen, and carbohydrate antigen 19-9 (CA19-9) 3 months before and after the diagnosis have been obtained from the electronic Hospital Information Management System. Neutrophil lymphocyte ratio (NLR) and platelet lymphocyte ratio (PLR) were calculated. In addition, the most recent computerized tomography (CT), magnetic resonance imaging, positron emission tomography (PET-CT), Ga68 DOTATATE PET CT, and endoscopic ultrasound findings of the patients before and 3 months after diagnosis were also recorded.

Fujinon endoscopy (Fujinon, Tokyo, Japan) and Fujinon EG-530U radial echoendoscope (Fujinon) were used for all endoscopy and endoscopic ultrasound evaluations. In patients with control endoscopy, the follow-up year interval and the number of endoscopy performed in this interval were calculated. Endoscopic biopsy localization, presence of polyp, polyp size (<1 cm or >1 cm), and the procedure performed (excision with forceps, excision with snare, or biopsy) were recorded.

Chromogranin A (immunohistochemistry) was studied in all endoscopic gastric biopsy tissues included in the study. It is almost impossible to detect simple/diffuse type hyperplasia at hematoxylin–eosin level alone. It can be detected incidentally in immunohistochemistry applied as a result of suspicion of more advanced neuroendocrine lesions.

Neuroendocrine cell hyperplasia definition and criteria for defining its histological subtypes are as follows:

- Simple/diffuse type: increase in a large number of gland structures individually or up to 3 cells,
- Linear type: neuroendocrine cells with linear, semi-linear, or crown-like arrangement,
- Micronodular type: neuroendocrine cell islands (100-150 µm/mean diameter of 1 gastric gland),
- Adenomatoid hyperplasia: it was determined as the presence of 5 or more micronodules close to each other between the glands.
- Although less than 500 microns, micronodules that tend to coalesce or contain stroma in the background or nodules that show microinvasion are called dysplasia. Neuroendocrine tumor was defined as the presence of nodular growth greater than 500 µm (0.5 mm) in diameter.^10^

The NECH regression criterion was the absence of NECH in 2 or more control endoscopy performed at different times. Only patients who did not have NECH in 1 control endoscopy were excluded from the analysis. Other histopathological findings such as the presence of atrophy, *H. pylori*, IM, paneth cell metaplasia, lymphocyte infiltration, neutrophil infiltration, and dysplasia were noted. Severity ratings were performed for atrophy, *H. pylori*, and IM as “+,” “++,” and “+++.”

### Statistical Analysis

All analyses were performed using the Statistical Package for the Social Sciences version 17.0 (SPSS Inc.; Chicago, IL, USA) statistical package program. In the descriptive statistics section, categorical variables were presented as numbers and percentages, and continuous variables were presented as mean ± SD and median (smallest-maximum value). Conformity of continuous variables to normal distribution was evaluated using visual (histogram and probability graphs) and analytical methods (Kolmogorov–Smirnov/Shapiro–Wilk tests). While the Mann–Whitney *U* test is used for comparison analysis between 2 groups for data that do not fit normal distribution, independent sample *t* test is used for comparison analysis between 2 groups for data that fit normal distribution; chi-square test was used to compare the quantitative values of independent groups. The independent mean was compared with the post-hoc Dunn test after the Kruskal–Wallis test. Statistical significance was defined as *P* < .05.

## Results

Of the 282 patients included in the study, 55.7% were female and 44.3% were male, and the mean age was 60.1 ± 14.2 years. Hashimoto’s thyroiditis was present in 34 patients (12.1%) and T1DM in 39 patients (13.8%), and both diseases were present in 7 patients (2.5%). The CAG rate in our study group was 65.2%. On the other hand, 27.5% of our patients with CAG + NECH had an accompanying autoimmune disease. There was no significant difference between the groups with and without CAG in terms of autoimmune disease *(P* = .62). Demographic information and the laboratory findings of the patients included in the study are elaborated in Tables 1 and 2.

The mean diagnostic endoscopy was 3.0 ± 2.3 years ago. Endoscopic findings were erythematous pangastritis in 107 patients (37.9%), polyps in 63 patients (22.3%), atrophic gastritis in 69 patients (24.5%), erosive gastritis in 19 patients (6.7%), ulceroerosive lesion in 11 patients (3.9%), and mucosal lesions in 2 patients. Raised eroded area was reported in 3 patients (1.4%), external pressure to the stomach in 1 patient (0.4%), and normal findings in 6 patients (2.1%). Histological subtypes of NECH and other histopathological findings are denoted in Table 3.

Neuroendocrine tumor developed in 12 (4.3%) of 282 patients with NECH after a mean of 36 months. The median endoscopic follow-up period of these patients was 24 months (minimum 3 months, maximum 132 months, and range 129 months), and the number of endoscopy performed during this period was 2.4 (minimum 2, maximum 6, and range 4). Up to 72.7% of the patients who developed NET on the basis of NECH had been using PPIs, 58.3% had atrophy, and 41.7% had *H. pylori*. The distribution in terms of PPI use, presence of atrophy, and presence of *H. pylori* was similar between the patient groups who developed and did not develop NET in the follow-up (*P* = .67, *P* = .59, and *P* = .12, respectively).

There was no significant difference between the 2 groups in terms of gender distribution and mean age of patients with and without NET (*P* = .68 and *P* = .14**)**. Hashimoto’s thyroiditis was similar between the groups (*P* = .29), and there was a significant difference in T1DM in favor of the group without NET (*P* = .03). While there was a no difference in favor of the non-NET group in terms of NSAIDs between the groups *(P* = .33), the distribution between the groups was similar in terms of other drugs (*P* = .32 and *P* = .67) (Table 1).

The most common reason for admission was dyspepsia (41.7%) in the NET (41.7%) and non-NET group (39.2%). There was no statistical significance between the 2 groups in terms of the presence of symptoms (*P* = .97), and iron deficiency was stated as an indication for endoscopy in all asymptomatic patients (Table 1). There was no significant difference between the laboratory values in the group with and without NET (Table 2).

Of the patients with NET, Ga68 DOTATATE PET CT was performed in 66.7% of the patients. Positive findings supporting the diagnosis were reported in 95% of Ga68 DOTATATE PET CT. In addition, Ga68 DOTATATE PET CT was performed in 1.5% of the patients without NET. There was a statistically significant difference between the groups with and without NET in favor of the group with NET in terms of Ga68 DOTATATE PET CT *(P* < .01).

There were gastric polyps (66.7%) in 8 patients in the NET group. Majority of the (66.6%) of the polyps were smaller than 1 cm, 16.7% were between 1 and 2 cm, and 16.7% were larger than 2 cm. In the group without NET, there were polyps in the stomach (17.3%) in 47 patients. Most of the (81.6%) polyps were smaller than 1 cm, 16.2% were between 1 and 2 cm, and 2.2% were larger than 2 cm. The presence of polyps was significantly different in favor of the NET group (*P* < .01). There was no significant difference between the 2 groups in terms of polyp size (*P* = .36), and the presence of polyps in the endoscopic appearance was prominent in patients with NET, and atrophic gastritis and erythematous pangastritis were predominant in patients without NET.

In terms of NECH histological subtypes, the linear + micronodular type was more common in both the non-NET group and the NET group. There was no statistical difference between the groups in terms of linear, micronodular, and linear + micronodular histological subtypes (*P* = .02, *P* = .06, and *P* = .61, respectively). The simple/diffuse type was statistically higher in the NET group than in the non-NET group (*P* = .008). *H. pylori* positivity and the presence of neutrophil infiltration were in favor of the non-NET group (*P* = .13 and *P* = .03), and the presence of dysplasia was significantly higher in favor of the NET group (*P* = .001) (Table 3).

In this study, there were 110 control endoscopy procedures: 12/12 (100%) in the NET group and 102/270 (37.7%) in the non-NET group. The mean endoscopic follow-up interval was 24 ± 21.6 months (3-132 months), and the total number of endoscopies per person was 2.4 ± 1.5.^1-9^ Nineteen of these patients (28.7%) had regression and 47 did not (71.3%).

In terms of all demographic data, no significant difference was found between the groups with and without regression of NECH (*P* = .13,* P* = .17, and *P* = .68) (Table 4). The sedimentation value was significantly higher in the group whose NECH did not regress (*P* = .02). In addition, there was no significant difference between the NLR and PLR rates between the 2 groups (*P* = .23 and *P* = .61) (Table 5). The endoscopic features of the patients whose NECH regressed and did not regress are shown in Table 6. No significant difference was found between the groups when the histological subtypes of the patients in both groups were compared (Table 6).

## Discussion

This is an important aspect as NECH is considered a pre-neoplastic lesion and can progress to NET. It was thought that the widespread use of PPIs, the increased awareness of pathologists on this issue, and the widespread use of IHC methods in the routine, especially in the dramatic increase in the diagnosis of NECH in the last 3 years, contributed to this awareness. Neuroendocrine cell hyperplasia and gNETs constitute a heterogeneous group of diseases that can show very different behaviors, ranging from relatively indolent growth of gastric carcinoids to the malignant attitude of appendiceal and hindgut carcinoids, requiring different recommendations for their follow-up and surgical treatment. Data on NECH are limited in the previous literature. In 1 study, the rate of progression from NECH to NET was 7.0% after a follow-up period of 90.1 months.^10^ In our series, NET developed at a rate of 4.3% (12/282) after an average of 36 months in the follow-up of the patients.

The mean age of the patients with NECH was 60.3 and the female/male ratio was 1.25 : 1; the mean age of the patients with NET was 55.7 and the female/male ratio was 1 : 1. According to the results of our study, patients with NECH were at an advanced age at the time of diagnosis, and NECH was more common in women than in men. Similarly, in a study revealing the relationship between PPI use and the development of NECH, the mean age of the patients was found to be 54 years.^11^ In another study dealing with patients with gNET, the mean age was found to be 58.2 years and the female/male ratio was 1.36 : 1.^12^ Female predominance can be attributed to exposure to environmental risk factors, genetic factors, or tumor-specific biological effects of sex hormones. At the same time, the fact that autoimmune atrophic gastritis, which is frequently associated with neuroendocrine cell pathologies, is more common in women may be a factor that explains this situation.

There was evidence that other autoimmune diseases, especially autoimmune thyroid disorders (AITDs) and T1DM, were seen more frequently with autoimmune CAG. In a study by Carabotti et al,^13^ the prevalence of other accompanying autoimmune diseases in patients with chronic autoimmune gastritis was reported to be approximately 40%. The CAG rate in our study group was 65.2%. There was an accompanying autoimmune disease in 27.5% of our patients with CAG + NECH. There was no significant difference between the groups with and without CAG in terms of autoimmune diseases (*P* = .62). In a retrospective study conducted by Vanoli et al,^10 ^an autoimmune disease was found to accompany 25.0% of the 100 patients with CAG + NECH. In another study, micronodular NECH developed in 6 of 40 patients with AITDs + autoimmune gastritis, linear + micronodular NECH developed in 1 patient (17.5%), and type 1 gNET was observed in 1 patient with micronodular NECH (2%-5%) after 39 months of follow-up.^14^ In our study, the frequency of Hashimoto’s thyroiditis was higher in the group with NET, and the frequency of T1DM was significant in favor of the group without NET (*P* = .03). In a retrospective study consisting of 111 patients with NET, Hashimoto’s thyroiditis was found in 36.9% of patients and 9.9% of DM patients.^15^ Similar to the literature, Hashimoto’s thyroiditis was observed more frequently as an autoimmune comorbidity than T1DM in our study. For this reason, it should be kept in mind that patients with autoimmune diseases such as Hashimoto’s thyroiditis and T1DM are at risk for the development of NECH and NET.

It is known that increased gastrin levels secondary to the hypoacidity caused by long-term use of PPIs can lead to NECH. In order to reveal the effect of PPI use on gastric histopathology, 16 studies were evaluated in a meta-analysis involving a total of 1920 patients. After long-term use of PPIs (at least 3 years), the rate of NECH has been reported to be between 7.8% and 52%, but NET has not been observed.^16^ In contrast, there is indirect evidence of NET development after PPI use. In a study in which 31 patients with gNET were evaluated, all other etiologies were excluded in 9.6% of the patients, and ultimately the development of NET was attributed to the use of PPIs. It has been interpreted that NET is a rare but serious side effect of chronic PPI use (>5-15 years).^17^ In our study, 59.9% of the cases with NECH were using PPIs. The rate of PPI usage was similar in patients with and without regression of NECH and in patients with and without NET. In the study of Jianu et al,^18^ it was observed that NECH could regress 3-20 months after the discontinuation of PPIs. However, in our study, we may have obtained different results because we obtained drug information only from e-prescription records, and therefore we could not obtain information about the dose and duration of exposure to patients and whether they used the drug regularly. In addition, the use of PPI in these patient groups may have been low, since there is a tendency to discontinue PPI in patients with NECH and/or NET in endoscopic biopsies.

In a retrospective study conducted by Vanoli et al^10^ on 100 patients with CAG + NECH, macrocytic anemia was detected in 7.0% of the patients. In our study, accompanying anemia was found in 47.3% of the patients with NECH, but there was no difference in the presence of anemia between the groups with and without NET or in which NECH regressed and did not regress. It was observed that anemia was not a predictive factor for the development of NET or regression of NECH in the background of NECH. However, in our study, the indication for endoscopy in asymptomatic cases was mostly anemia, which ultimately led to the detection of NECH and NET in asymptomatic cases.

No study was found in the literature on the laboratory findings of patients diagnosed with NECH. The sedimentation level was significantly higher in the group whose NECH did not regress (*P* = .02). There was no significant difference between the groups with and without NET and between groups with and without regression of NECH when other laboratory parameters were compared. Neutrophil lymphocyte ratio and PLR are being investigated as helpful inflammatory markers in predicting prognosis in NETs as well as in many solid tumors. There are studies supporting that NLR and PLR in gastroenteropancreatic neuroendocrine tumors (GEP)-NETs are positively correlated with tumor grade.^19^ In our study, the rates of NLR and PLR were similar in the group with and without NETs.

It is important to recognize the endoscopic features of NECH and gNETs because patients are usually asymptomatic and are incidentally detected during endoscopy for different reasons. There is no specific endoscopic finding related to NECHs.^20^ In a study conducted on 666 patients with gastric polyps, it was reported that NET was detected with a rate of 3.4% by polyp biopsies.^21^ In our study, polyps were detected in 17.3% and 66.7%, respectively, of patients with endoscopic NECH and NET (*P* < .01), while both groups were similar in terms of other endoscopic findings. In conclusion, the presence of polypoid appearance due to NECH in endoscopy was a predictive factor for NETs.

Radiological and nuclear medicine imaging methods do not have specific findings supporting the diagnosis of NECH. In NETs, these methods are used to localize the tumor and detect metastases and mesentery invasion. Geijer and Breimer^22^ reported that the sensitivity of Ga68 DOTATATE PET CT in the imaging of NETs varies between 70% and 100% in their meta-analysis, in which they included a total of 2105 patients from 22 studies. In our study, positive findings supporting the diagnosis were reported in 95% of patients with gNET who had Ga68 DOTATATE PET CT withdrawn.

Atrophy and IM develop in response to chronic mucosal inflammation, and as a result, acid secretion decreases with loss of principal cells. Enterochromaffin-like cell stimulation occurs with G cell hyperplasia in response to decreased acidity.^23^ Therefore, it is expected that these 2 histopathological findings are frequently associated with NECH and NET. In our study, 64.8% and 58.3% of the patients with NECH and NET had atrophy and 69.2% and 75% had IM, respectively, and there was no difference between the groups. In addition, there was no difference between groups in which NECH regressed and those that did not (*P* = .35 and *P* = .74). In a study conducted with 104 patients with NECH or NET at the Hacettepe University Medical Faculty Hospital, concomitant atrophy + IM was found in the surrounding mucosa in 84% of the patients and at rates similar to ours.^24^

Patients with oxyntic mucosal atrophy due to *H. pylori* infection are hypergastrinemic due to decreased gastric acidity. Gastrin both stimulates histamine release from ECL cells and has a trophic effect on cells.^25,26^ In a meta-analysis of 16 studies (1920 patients), a statistically significant increased risk of developing NECH was observed in *H. pylori*-positive patients compared to *H. pylori*-negative patients [odds ratio: 2.45 (95% CI: 1.47-4.10), *P* = .0006]; however, no evidence of neoplastic change was found.^16^ In our study, *H. pylori* was positive in 62.2% of patients with NECH. Patients with and without regression of NECH were similar in terms of *H. pylori* positivity rate. Although type I gNETs are frequently seen in autoimmune gastritis, they may also rarely occur in the setting of atrophic gastritis secondary to chronic *H. pylori* infection.^27^ Five cases of NET infected with *H. pylori* without CAG or ZES have been reported in Japan.^28^ However, in our study, there was a statistically significant difference in the presence of *H. pylori* compared to the group without NET. This suggests that non-*H. pylori* factors are important in the development of NECH and NET. The trophic effect of gastrin, which is thought to cause NECH, is not the only factor that increases tumor growth. Loss or incorrect expression of tumor-suppressor genes, Reg-1 protein expression, dysregulation of somatostatin receptor genes, or *H. pylori* or autoimmune chronic gastritis can alter the effects of PPIs in different individuals.^29^

Dysplasia is a lesion defined as “atypical hyperplasia” and causes an approximately 20-fold increase in risk for the development of type 1 gNETs.^7^ In the study conducted by Vanoli et al,^10^ dysplasia was reported in 20% of patients with NECH at the time of diagnosis or developed during the follow-up period. In our study, dysplasia detected in the pathological examination of lesions with NECH was found at a rate of 16.7% (2/12) in the group with NETs, which was significantly higher than that in the group without NET (*P* = .01).

In the literature, there was no recommendation with a high level of evidence regarding the frequency of follow-up of cases with NECH. In our study, it was observed that NET developed in 12 (4.3%) of 282 patients diagnosed with NECH in an average of 36 months during follow-up. The median follow-up period of these patients was 24 months (3 months-132 months), and the mean number of endoscopy performed during this period was 2.4.^1-9^ Endoscopy was performed 6 times in 11 years for the patient with the longest follow-up period. In a similar study conducted at the Hacettepe University Faculty of Medicine Hospital, of 104 patients, 45 were diagnosed with NECH (43%), 49 with type 1 NETs (47%), 7 with type 2 NETs (7%), and 3 with type 3 NETs (3%) in their endoscopic biopsies. It was determined that 30 patients were followed up with endoscopic biopsy after diagnosis, and the mean follow-up period was 25 months (5 months-7 years). A total of 17 endoscopic biopsies were performed in 7 years for the patient with the longest follow-up period.^24^ There was not enough data regarding the necessity of endoscopic follow-up and endoscopic follow-up intervals in patients with NECH. It may be possible to identify especially high-risk patients and to develop appropriate screening programs by revealing the factors that play a role in the development of NECH and NET.

The main limitation of this study could be attributed to its retrospective nature. Other limitations could be the insufficient follow-up time and the lower number of biopsies than recommended in the Sydney protocol for endoscopic sampling.

The rate of NET development in the background of NECH has been 4.3% on average in 36 months. No significant difference was detected in this transformation in terms of demographic findings, smoking, alcohol consumption, drug use, initial symptoms, laboratory findings, imaging and endoscopy findings, and histopathological features investigated to predict the progression from NECH to NET. Among the data examined within the scope of this study, 2 factors predicting the progression from NECH to NET can be elaborated as the presence of polyps in endoscopy and dysplasia in histopathological examination. There is a need for more comprehensive prospective studies on this subject.

## Figures and Tables

**Table 1.** Comparison of Demographic and Clinical Characteristics of Cases With and Without NETs

	With NETs (n = 12)	Without NETs (n = 270)	Total (n = 282)	*P*
Age	55.7 ± 10.7	60.3 ± 14.3	60.1 ± 14.2	.14
Sex				
Female	6/12 (50.0)	151/270 (55.9)	157/282 (55.7)	.68
Male	6/12 (50.0)	119/270 (44.1)	125/282 (44.3)	
Smoking	0/3 (0.0)	46/165 (27.8)	46/168 (27.3)	.03
Alcohol use	0/3 (0.0)	31/159 (19.4)	31/162 (19.1)	**.001**
Family history of gastric cancer	0/2 (0.0)	9/162 (5.5)	9/164 (5.4)	.12
Comorbid diseases				
Hashimoto’s thyroiditis	3/12 (33.3)	38/270 (14.0)	41/282 (15.1)	.29
Type 1 DM	0/12 (0.0)	47/270 (17.4)	47/282 (16.6)	.03
PPI use	8/11 (72.7)	145/242 (59.9)	153/253 (60.4)	.67
Aspirin use	1/11 (9.0)	69/238 (28.9)	70/249 (28.1)	.32
NSAID use	2/11 (18.1)	91/235 (38.7)	93/244 (38.1)	.33
Presenting symptom				
Asymptomatic	4 (33.3)	87 (32.2)	91/282 (32.2)	.97
Dyspepsia	5 (41.7)	106 (39.2)	111/282 (39.3)	
Stomach ache	2 (16.7)	53 (19.6)	55/282 (19.6)	
Weight loss	–	7 (2.6)	7/282 (2.5)	
Other	1 (8.3)	17 (6.4)	18/282 (6.4)	

Statistical significance was defined as *P* < .05. DM, diabetes mellitus; NET, neuroendocrine tumor; NSAID, non-steroidal anti-inflammatory drug; PPI, proton pump inhibitor.

**Table 2.** Comparison of Laboratory Parameters of Cases With and Without NETs

	With NET (n = 12)	Without NET (n = 270)	Total (n = 282)	*P*
Hemoglobin, g/dL	12.5 ± 2.3	12.4 ± 1.9	12.4 ± 1.9	NS
Hematocrit, (%)	37.7 ± 5.8	37.6 ± 5.4	37.6 ± 5.4	NS
Leukocyte, 10^3^/µL	6.9 ± 1.9	7.4 ± 4.4	7.4 ± 4.3	NS
Neutrophil, 10^3^/µL	3.6 ± 1.2	4.7 ± 3.4	4.7 ± 3.3	NS
Lymphocyte, 10^3^/µL	2.1 ± 0.3	2.0 ± 1.5	2.0 ± 1.4	NS
Platelet, 10^3^/µL	219 ± 83	258 ± 83	257 ± 84	NS
LDH, U/L	201	185.0	185.0	NS
ESR, mm/h	29.0	21.5	26.0	NS
CRP, mg/L	2.8	2.6	5.2	NS
CA19-9 U/mL	11.0	7.5	7.8	NS
CEA, ng/mL	2.9	2.4	2.2	NS

CA19-9, carbohydrate antigen 19-9; CEA, carcinoembryonic antigen; CRP, C-reactive protein; ESR, erythrocyte sedimentation rate; LDH, lactate dehydrogenase; NET, neuroendocrine tumor; NS, not significant.

**Table 3.** Comparison of Endoscopic and Histopathological Features of Cases with and Without NETs

	With NET (n = 12)	Without NET (n = 270)	Total (n = 282)	*P*
Polyps	8 (66.7)	47 (17.3)	55 (19.4)	**<.01**
NECH histological subtype				
Linear	0 (0.0)	22 (8.2)	22 (7.8)	.02
Micronodular	2 (16.7)	37 (13.7)	39 (13.8)	.06
Linear + micronodular	6 (50)	136 (50.4)	142 (50.4)	.61
Simple/diffuse type	4 (33.3)	75 (27.7)	79 (28.0)	**.03**
Atrophy	7 (58.3)	177 (64.8)	184 (65.2)	.59
Degree				
+	0 (0.0)	53 (30.2)		
++	2 (28.5)	74 (46.8)		
+++	5 (71.5)	40 (23.0)		
*H.* *pylori* (+)	5 (41.7)	170 (62.2)	175 (62.1)	.03
Degree				
+	2 (40.0)	46 (27.3)		
++	2 (40.0)	104 (61.1)		
+++	1 (20.0)	20 (11.6)		
IM (+)	9 (75.0)	187 (69.2)	196 (69.5)	.47
Degree				
+	4 (44.5)	70 (37.4)		
++	2 (22.2)	84 (44.9)		
+++	3 (33.3)	33 (17.7)		
Paneth cell metaplasia	2 (16.7)	18 (6.6)	20 (7.1)	.20
Lymphocyte infiltration	12 (100)	249 (92.2)	261 (92.5)	.38
Neutrophil infiltration	2 (16.7)	127 (47.0)	129 (45.8)	.03
Dysplasia	2 (16.7)	3 (1.1)	5(1.8)	**.001**

Statistical significance was defined as *P* < .05. *H. pylori*, *Helicobacter pylori*; IM, intestinal metaplasia; NECH, neuroendocrine cell hyperplasia; NET, neuroendocrine tumor.

**Table 4.** Comparison of Demographic and Clinical Characteristics of Patients With and Without Regression of NECH

	With Regression of NECH (n = 19)	Without Regression of NECH (n = 47)	*P*
Age	60.6 ± 9.6	61.7 ± 11.1	.70
Sex			.37
Female	12/19 (63.1)	24/47 (51.0)	
Male	7/19 (36.9)	23/47 (49.0)	
Smoking	5/14 (35.7)	3/21 (14.2)	.13
Alcohol use	3/14 (21.4)	1/18 (5.5)	.17
Family history of gastric cancer	1/11 (9.0)	1/19 (5.2)	.68
Comorbid diseases	5/19 (26.3)	9/47 (19.1)	.52
PPI use	10/17 (58.8)	25/45 (55.5)	.81
Aspirin use	4/17 (23.5)	11/45 (24.4)	.99
NSAID use	6/17 (35.2)	12/45 (26.6)	.54
Presenting symptom			
Asymptomatic	1/19 (5.3)	16/47 (34.0)	**.02**
Dyspepsia	13/19(68.4)	22/45 (46.8)	
Stomach ache	4/19 (21.0)	7/45 (14.9)	
Weight loss	1/19(5.3)	–	
Other	–	2/47 (4.3)	

Statistical significance was defined as *P* < .05. NECH, neuroendocrine cell hyperplasia; NSAID, non-steroidal anti-inflammatory drug; PPI, proton pump inhibitor.

**Table 5.** Comparison of Laboratory Parameters of Patients With and Without Regression of NECH

	With Regression of NECH (n = 19)	Without Regression of NECH (n = 47)	*P*
Hemoglobin, g/dL	12.7 ± 1.6	12.3 ± 1.9	NS
Hematocrit, %	38.7 ± 4.6	37.2 ± 5.6	NS
Leucocyte, 10^3^/µL	6.9 ± 1.4	7.0 ± 1.9	NS
Neutrophil, 10^3^/µL	4.1 ± 1.0	3.8 ± 1.4	NS
Lymphocyte, 10^3^/µL	2.0 ± 0.7	2.2 ± 1.1	NS
Platelet, 10^3^/µL	236 ± 70	244 ± 78	NS
LDH, U/L	180.2 ± 20.7	187.5 ± 41.7	NS
ESR, mm/h	4.0	30.0	**.02**
CRP, mg/L	4.2	2.1	NS
CA19-9 U/mL	16.6	7.4	NS
CEA, ng/mL	2.5	2.6	NS

Statistical significance was defined as *P *< .05. CA19-9, carbohydrate antigen 19-9; CEA, carcinoembryonic antigen; CRP, C-reactive protein; ESR, erythrocyte sedimentation rate; LDH, lactate dehydrogenase; NECH, neuroendocrine cell hyperplasia; NS, not significant.

**Table 6.** Comparison of Endoscopic and Histopathological Features of Patients With and Without Regression of NECH

	With Regression of NECH (n = 19)	Without Regression of NECH (n = 47)	*P*
Endoscopic appearances			
Gastric polyp	7 (36.7)	22 (46.8)	.39
Atrophic gastritis	3 (15.8)	12 (25.6)	
Erythematous gastritis	6 (31.6)	9 (19.2)	
Erosive gastritis	1 (5.3)	–	
Ulceroerosive lesion	2 (10.6)	1 (2.1)	
Raised eroded area from the mucosa	–	1 (2.1)	
Normal	–	2 (4.2)	
NECH histological subtype			
Linear	4 (21.0)	9 (19.1)	.99
Micronodular	2 (10.5)	10 (21.4)	.48
Linear + micronodular	12 (63.2)	23 (48.9)	.29
Simple/diffuse type	1 (5.3)	5 (10.6)	.49
Atrophy	12 (63.1)	35 (74.4)	
Degree			
+	6 (50.0)	5 (14.4)	.35
++	4 (33.3)	15 (42.8)	
+++	2 (16.7)	15 (42.8)	
*H.* *pylori* (+)	16 (84.2)	31 (65.9)	.13
Degree			
+	5 (31.2)	10 (32.2)	
++	9 (56.2)	18 (58.0)	
+++	2 (12.6)	3 (9.8)	
IM (+)	14 (73.6)	37 (78.7)	.74
Degree			
+	2 (14.3)	16 (43.2)	
++	8 (57.1)	14 (37.8)	
+++	4 (28.6)	7 (19.0)	
Paneth cell metaplasia	2 (10.5)	6 (12.8)	.99
Lymphocyte infiltration	19 (100)	44 (93.6)	.26
Neutrophil infiltration	10 (52.6)	18 (38.3)	.28
Dysplasia	–	3 (6.4)	–

IM, intestinal metaplasia; *H. pylori*, *Helicobacter pylori*; NECH, neuroendocrine cell hyperplasia.
